# Nucleotide Transport and Metabolism in Diatoms

**DOI:** 10.3390/biom9120761

**Published:** 2019-11-21

**Authors:** Ansgar Gruber, Ilka Haferkamp

**Affiliations:** 1 Institute of Parasitology, Biology Centre, Czech Academy of Sciences, Branišovská 1160/31, 370 05 České Budějovice, Czech Republic; 2 Pflanzenphysiologie, Technische Universität Kaiserslautern, 67663 Kaiserslautern, Germany; haferk@rhrk.uni-kl.de

**Keywords:** endosymbiosis, plastid, photosynthesis, adenosine triphosphate (ATP), transport, evolution, synthetic biology

## Abstract

Plastids, organelles that evolved from cyanobacteria via endosymbiosis in eukaryotes, provide carbohydrates for the formation of biomass and for mitochondrial energy production to the cell. They generate their own energy in the form of the nucleotide adenosine triphosphate (ATP). However, plastids of non-photosynthetic tissues, or during the dark, depend on external supply of ATP. A dedicated antiporter that exchanges ATP against adenosine diphosphate (ADP) plus inorganic phosphate (Pi) takes over this function in most photosynthetic eukaryotes. Additional forms of such nucleotide transporters (NTTs), with deviating activities, are found in intracellular bacteria, and, surprisingly, also in diatoms, a group of algae that acquired their plastids from other eukaryotes via one (or even several) additional endosymbioses compared to algae with primary plastids and higher plants. In this review, we summarize what is known about the nucleotide synthesis and transport pathways in diatom cells, and discuss the evolutionary implications of the presence of the additional NTTs in diatoms, as well as their applications in biotechnology.

## 1. Introduction

Photosynthetic primary production is at the base of the vast majority of ecosystems in the world [1]. In the oceans, diatoms are a particularly dominant group of eukaryotic phytoplankton, by species numbers as well as by their combined productivity [1,2,3]. Like in all photosynthetic eukaryotes, their photosynthetic cell organelles, the plastids, go back to cyanobacterial ancestors, which were taken up by eukaryotes in an endosymbiosis that led to the evolution of the organelle [4,5,6,7]. Plastids that directly evolved from cyanobacteria via prokaryote-eukaryote endosymbiosis are called primary plastids. Plastids found in all major groups of eukaryotes appear to be monophyletic; however, the phylogenies of the plastids are in many cases different from the ones of the host cells, which means that many groups of eukaryotes must have acquired their plastids independently, and from eukaryotic donors [3,4,8,9]. In addition to the widespread primary plastids of the Archaeplastida (red algae, glaucocystophyta and green algae, including higher plants), only one other example of a plastid acquisition from cyanobacteria is known, the one in the Cercazoan *Paulinella chromatophora* [10]. Diatom plastids represent so-called complex plastids, which arose via eukaryote-eukaryote endosymbiosis [4,7,11,12]. They are surrounded by four envelope membranes, the outermost of which is continuous with the endoplasmic reticulum (the cER, for chloroplast endoplasmic reticulum), and the innermost two membranes apparently correspond to the inner and outer envelope membranes of primary plastids [11,12]. The second membrane from the outside, together with the second membrane from the inside, defines the periplastidic space, a sub-compartment of the diatom plastid in which cell biological processes and a couple of metabolic reactions take place [13,14,15,16].

**Figure 1:**
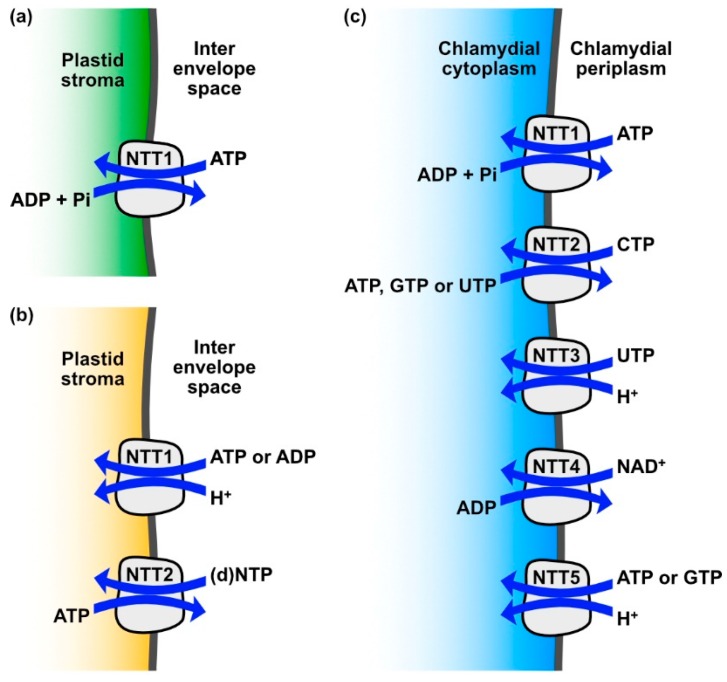
Transport activities of various nucleotide transporters (NTTs): (**a**) plant NTTs exchange adenosine triphosphate (ATP) against adenosine diphosphate (ADP) + inorganic phosphate (Pi) and thereby provide energy to the plastid when photophosphorylation is limited or not possible; (**b**) diatom NTT1 and 2 isoforms, in combination, facilitate net transport of a broad spectrum of nucleotides into the stroma ((d)NTP stands for the tested substrates cytidine triphosphate (CTP), guanosine triphosphate (GTP), deoxy-CTP (dCTP), ATP, uridine triphosphate (UTP), deoxy-GTP (dGTP), deoxy-ATP (dATP), thymidine triphosphate (TTP)); (**c**) in the environmental chlamydiae strain *Protochlamydia amoebophila*, NTT1 enables energy parasitism similarly to plant NTTs, whereas NTT2, 3 and 5 enable net nucleotide provision similar to NTT1 and 2 from diatoms and NTT4 allows the intracellular bacterium to extract nicotinamide adenine dinucleotide (NAD^+^) from the host metabolism.

Cyanobacteria store the photosynthesized carbohydrates in the bacterial cytoplasm in the form of glycogen and use these stores for the generation of energy via respiration, which allows them to tide over conditions in which photosynthesis is not possible, for instance in the absence of light [17]. In contrast, in the majority of photosynthetic eukaryotes, storage carbohydrates are located outside of the plastid stroma and hence outside of the former cyanobacterial cytosol. However, green algae and plants are a notable exception, because their starch metabolism has secondarily been re-allocated to the plastid [18,19]. Consequently, whenever photosynthesis is insufficient to meet the ATP demand, the plastid needs to be supplied with this energy currency from other sources. The corresponding ATP production can take place in mitochondria (through respiration), or in the cytosol (through substrate-level phosphorylation). In photosynthetic eukaryotes, plastidic ATP uptake is catalyzed by specific solute transport proteins called nucleotide transporters (NTTs) [20,21,22]. These NTTs are present in the inner envelope membrane, where they act as antiporters, exchanging ATP against adenosine diphosphate (ADP) plus inorganic phosphate (Pi) [20,23] (Figure 1a).

Like most photosynthetic eukaryotes, also diatoms store carbohydrates outside of the plastids [24]; however, their metabolism, as well as many other features, including the function of NTTs, turn out to be quite unique compared to other photosynthetic eukaryotes [25,26,27]. In the following, we will compare the role of diatom NTTs with that of NTTs found in other organisms. We will discuss what this tells us about the evolution of primary plastids, the evolution of complex plastids in diatoms, and we will summarize recent advances in synthetic biology, which were enabled by a diatom NTT.

## 2. Nucleotide Synthesis in Diatom Cells

Nucleotides are important for the transfer and conversion of chemical energy in the cell, most prominently in the form of the energy currency ATP. Furthermore, nucleotides also represent phytohormone precursors, may act as signal molecules, and are the building blocks for DNA and RNA [28]. Therefore, cellular growth requires the de-novo synthesis of nucleotides. Purine and pyrimidine nucleotides are synthesized in distinct pathways. While in purine biosynthesis, the whole pathway takes place on the scaffold of the ribose phosphate (provided from ribose-5-phosphate via 5-phosphoribosyl-1-pyrophosphate (PRPP)) [28], pyrimidine biosynthesis starts with the formation of carbamoyl phosphate from bicarbonate and glutamine (and the conversion of two ATP to two ADP+Pi) [28,29]. The later steps of this pathway, however, also involve the attachment of ribose-5-phosphate via PRPP by the enzyme uridine-5′-monophosphate synthase (UMPS) [28,29]. In plants, the ribose-5-phosphate used for nucleotide biosynthesis is usually produced in the oxidative pentose phosphate pathway in the plastid [30], and also the complete purine biosynthesis and several steps of the pyrimidine synthesis take place within this organelle [28,31]. It is therefore remarkable that enzymatic studies [32], genome analyses [14,33], and targeting experiments [14] have shown that the key reactions of the oxidative pentose phosphate pathway are restricted to the cytosol in diatoms (although there are plastid targeted isoforms of transketolase and ribose-5-phosphate isomerase, which most likely provide ribulose-5-phosphate to the Calvin cycle in an anaplerotic reaction [14,33]).

Even more remarkable than the absence of the oxidative pentose phosphate pathway is the absence of a carbamoyl phosphate synthase from diatom plastids, which was discovered in the first whole genome-sequencing project for a diatom conducted by Armbrust et al. [34]. This project led to the identification of a urea cycle in the diatom *Thalassiosira pseudonana*, which is an unexpected finding in its own right [34,35]. In the urea cycle, a mitochondrial carbamoyl phosphate synthase that uses bicarbonate and ammonia (and the chemical energy from two ATPs that are hydrolysed to two ADP+Pi) catalyzes the formation of carbamoyl phosphate. The genome of *T. pseudonana* contains two copies for this type of carbamoyl phosphate synthase, one mitochondrial and one cytosolic [34]. Armbrust et al. commented the absence of a plastid targeted glutamine dependent carbamoyl phosphate synthase in *T. pseudonana* with the statement that “this observation raises the intriguing question of how pyrimidines are transported across the four plastid membranes” [34].

Following publication of the second genome of a diatom (*Phaeodactylum tricornutum*) [36], and using the characteristic sequence motif found in the pre-sequences of plastid targeted proteins of diatoms [37,38], Ast et al. [39] analyzed the intracellular distribution of the purine biosynthesis pathways in *T. pseudonana* and *P. tricornutum*, as well as the pyrimidine biosynthesis pathway in *P. tricornutum*, and found that most reactions of both nucleotide biosynthesis pathways are catalyzed in the cytosol of diatoms, with only a few intermediary steps that might occur at the cytosolic face of the mitochondrial membrane. Cytosolic location of the pyrimidine biosynthesis in diatoms is furthermore supported by the successful complementation of the *P. tricornutum* UMPS with the human UMPS, which did not require the addition of a targeting signal to the human (cytosolic) enzyme [40].

## 3. Nucleotide Transporters in Diatoms

The intracellular location of the nucleotide biosynthesis pathways in diatoms suggested the need for altered nucleotide transport capacities of their plastids when compared to those of higher plants, green, red and glaucophytic algae. In search for possible plastidic nucleotide uptake systems, Ast et al. screened the genomes of *T. pseudonana* and *P. tricornutum* and found an unusual large number of putative NTT coding sequences (eight in *T. pseudonana* and six in *P. tricornutum*, vs. one or two isoforms in other algae or plants) [39], with sizes ranging from 57 kDa to 92 kDa, and 7–11 predicted trans membrane helices (Table 1).

Due to the continuity of the cER with the ER of the cell, targeting of proteins to the plastids starts with signal peptide mediated co-translational import into the ER [12]. The signal peptides of ER and plastid proteins are interchangeable [38]. However, plastid proteins further possess a transit peptide, and they can be recognized by a distinct sequence motif at the signal peptide cleavage site, which, in reference to its one letter code amino acid sequence is called “ASAFAP”-motif [37,38]. This motif facilitates the prediction of diatom plastid proteins by manual sequence evaluation [33,51], or by prediction software [46,52]. In addition to proteins with plastid targeting pre-sequences consisting of signal peptides, transit peptides and “ASAFAP”-motif at the signal peptide cleavage site, sequences with signal and transit peptides but without “ASAFAP”-motif are also found, and these proteins are thought to be targeted to the periplastidic space [14,15,16,53,54].

According to Ast et al. [39], the NTTs identified in the two investigated diatom genomes show a variety of pre-sequence patterns. Either no N-terminal pre-sequences (*Tp*NTT4, *Tp*NTT5, *Tp*NTT7, *Tp*NTT8, *Pt*NTT3, *Pt*NTT4, *Pt*NTT5), simple endoplasmic reticulum (ER) signal peptides (*Tp*NTT2, *Pt*NTT2), bipartite targeting signals including “ASAFAP”-motif (*Tp*NTT1, *Pt*NTT1, *Pt*NTT6), or targeting pre-sequences that resemble those of proteins targeted to the periplastidic space (*Tp*NTT3 *Tp*NTT6) were distinguished [39]. However, based on alignments with bacterial NTTs, Major et al. identified N-terminal elongations in all these diatom NTTs; the function of these elongations as targeting peptides remains to be shown [41]. We tested the presence of plastid and mitochondrial targeting signals with the diatom plastid protein prediction program ASAFind [46] and with the secretory pathway and mitochondrial pre-sequence prediction program TargetP-2.0 [47], which were not available during Ast et al.’s previous analyses. The results of ASAFind support the findings of Ast et al. [39], and TargetP-2.0 did not predict any additional mitochondrial transit peptide in the NTTs of *P. tricornutum* and *T. pseudonana* (Table 1). Very little is known about the signals for targeting of a protein to the cytoplasmic membrane [55]. However, since it has been shown in radiolabelling experiments with [^3^H]-thymidine that diatoms can take up thymidine from the environment [56], it appears possible that some of the diatom NTTs are targeted to the cytoplasmic membrane to facilitate the uptake of dissolved organic nitrogen.

Green fluorescent protein (GFP) based targeting experiments have so far been conducted with three diatom NTTs: *Tp*NTT1 and *P*tNTT1 were localized to the innermost plastid envelope [39], *Tp*NTT2 and *Pt*NTT2 are present in the plastid envelope and/or endoplasmic reticulum membranes [39], and *Pt*NTT5 is found in the endoplasmic reticulum, including the cER [42]. In the case of *Pt*NTT5, the targeting depends on the location of the GFP reporter (fused either N-terminal or C-terminal), and Chu et al. [42] concluded from a series of truncation and fusion experiments that accessibility of the C-terminus is important for the ER localization of the protein, while the N-terminus may be altered for instance by fusion of GFP. Interestingly, the alignment performed by Major et al. [41], nevertheless, shows an N-terminal extension of this protein compared to the conserved NTT domain. Independently from GFP fusion experiments, proteomic analyses of plastid fractions of *T. pseudonana* provided evidence for the plastid association of *Tp*NTT1 and *Tp*NTT3 [43].

The most recent phylogenetic analyses show that except *Tp*NTT1 and *Pt*NTT1, which cluster with other plant and algal NTTs, the diatom NTTs are deeply divergent, with similar branch lengths to the plant NTTs as to NTTs from intracellular bacteria (*Rickettsiales*), or intracellular eukaryotic parasites (Microsporidia) [41,42].

Despite the sequence similarity of *Tp*NTT1 and *P*tNTT1 to the ATP/ADP+Pi antiporters of plants and other algae, their actual transport properties differ substantially. NTT1 of *T. pseudonana* and *P. tricornutum* do not act as antiporters, but mediate a proton driven net import of adenosine nucleotides [39] (Figure 1b, Table 2). This demonstrates that the transport properties of NTTs cannot be predicted by amino acid sequence similarity. Currently, the only known sequence feature that can be related to a substrate interaction is the conserved phosphate binding site of ATP/ADP antiporters [23]. The NTT2 isoforms of *T. pseudonana* and *P. tricornutum* represent antiporters, but exchange ATP against a large substrate spectrum of other nucleotides and desoxynucleotides [39]. Taken together, diatom NTT1 and NTT2 may form a system reminiscent of the systems described in intracellular bacteria that parasitize energy and nucleotides from the host cells [39] (Figure 1c). NTT5 of *P. tricornutum* (which has no orthologue in *T. pseudonana*) is an antiporter that exchanges adenine nucleotides against guanine nucleotides or against desoxy-ATP [42].

In general, NTTs are not widespread proteins. NTTs consisting of just one single NTT domain are only known from intracellular bacteria (*Chlamydiae* and *Rickettsiales*), photosynthetic eukaryotes, Microsporidia, and from a handful of other pro- and eukaryotes, intracellular as well as free living [41]. NTT domains were, however, also identified in fusions with HEAT-repeat and cyclic nucleotide binding domains [41].

Microsporidia are a group of eukaryotic obligate intracellular parasites [62] and their NTTs form a homologous and monophyletic group [41,60], although their function differs between the organisms characterized so far. *Encephalitozoon cuniculi* has four NTT isoforms, all *E. cuniculi* NTTs transport ATP/ADP in both directions; however, they differ in their intracellular locations. *Ec*NTT3 is found on mitosome membranes and is proposed to deliver energy to the mitochondrial relict. The remaining NTT isoforms are apparently present in the plasma membrane of the intracellular parasite [63]. By contrast, in *Trachipleistophora hominis,* all four NTT isoforms were detected in the plasma membrane [59]. While *T. hominis* NTTs 1-3 act as ATP/ADP or guanosine triphosphate/guanosine diphosphate (GTP/GDP) antiporters that also transport nicotinamide adenine dinucleotide (NAD^+^, NADH), NTT4 enables net import of ATP or GTP, in symport with protons [60]. Taken together, the plasma membrane located micorsporidial NTTs facilitate energy parasitism from the host cell, as well as purine nucleotide uptake from their hosts [60]. Microsporidial NTTs do not transport pyrimidine nucleotides; however, microsporidia have also lost the ability to synthesize pyrimidines, so the existence of a so far unknown transport system for pyrimidine nucleotides in microsporidia is postulated [59,60,64].

Since the NTT1 isoforms of diatoms do not catalyze energy uptake via ATP/ADP exchange, the question how energy is provided to diatom plastids in the dark (the function of other plant NTTs) is currently unanswered. It is imaginable that some of the so far uncharacterized NTTs fulfill this function. In this context, it is important to mention that there is growing evidence of a highly efficient redox and energy shuttling between diatom plastids and mitochondria. The transfer of reducing equivalents from the plastid to mitochondria may help prevent the formation of reactive oxygen species at the photosystems when excessive excitation energy is absorbed [35,65]. Moreover, mitochondrial energy (ATP) generated via respiration is used to drive photosynthetic carbon fixation in the plastid [65]. How this energetic coupling of photosynthesis and respiration is achieved is currently unknown [65]. Regarding the possible involvement of NTTs in the redox/energy transfer, two scenarios are possible. First, the diatom NTTs might be directly involved in this energy and redox shuttling. Interestingly, in the intracellular bacterium *Protochlamydia amoebophila*, an NTT-type transporter was shown to mediate the translocation of NAD^+^ in exchange with ADP [58]. Therefore, the biochemical capacity of an NTT to exchange reducing equivalents and adenine nucleotides has already been described. In addition, other known systems—a glycerol phosphate shuttle or a malate-aspartate shuttle—might contribute to the redox and energy shuttling. Alternatively, so far unknown enzymatic or structural systems might be involved in the redox and energy shuttling between diatom plastids and mitochondria.

Independent of the nature of the system used for energy translocation, its existence has enabled the evolution of the NTT1 proteins of diatoms away from their previously essential function as ATP/ADP + Pi exchangers. The new functions, such as the net transport of de-novo synthesized nucleotides, might then have allowed the loss of plastidic nucleotide biosynthesis. With respect to the hypothesis about so far unknown energy/redox shuttling systems, it also appears relevant that *P. tricornutum* apparently does not possess a malate-aspartate shuttle for the regeneration of NAD^+^ in the cytosol, as can be concluded from the absence of a cytosolic malate dehydrogenase [66]. Furthermore, the flexibility of adjusting ATP/reduction equivalent ratios and choosing between NAD^+^ and nicotinamide adenine dinucleotide phosphate (NADP^+^) in *P. tricornutum* mitochondria is increased, due to the presence of an Entner–Doudoroff pathway in the mitochondria [67], and due to the presence of a full set of enzymes for the second half of glycolysis in the mitochondria [68].

## 4. Evolutionary Implications of the Distribution of NTTs across the Tree of Life

NTTs with ADP/ATP antiporter activity are important for the maintenance of plastid metabolism in the absence of light energy and thus for photosynthetic ATP production in all photosynthetic eukaryotes. Interestingly, their evolutionary origin can neither be traced back to cyanobacteria (cyanobacteria, however, do possess NTT-HEAT domain fusion proteins [41]), nor to the eukaryotic non-photosynthetic ancestor of algae and plants with primary plastids. This gave rise to hypotheses of horizontal transfer of NTT genes from ancestors of infectious intracellular bacteria to the ancestor of photosynthetic eukaryotes during or preceding endosymbiosis with the plastid ancestor, known as the “menage a trois” hypothesis [6,69,70]. This hypothesis is based on the manipulation of the host glycogen metabolism by extant chlamydiae, and on phylogenies of a number of genes. While this hypothesis offers an elegant explanation of the mechanism that locked the cyanobacteria to the host cell, it is highly disputed due to the uncertainties of single gene phylogenies and due to the difficulties of inferring the directions of horizontal gene transfers, or even the existence of the postulated horizontal gene transfers (compare views presented in [71,72] vs. [73]).

One aspect of the “menage a trois” hypothesis is that it postulates that chlamydial NTTs allowed the cyanobacteria to persist in the host cell after they lost the ability to accumulate and consume their own glycogen [70]. Here, the patchy distribution of NTTs in the tree of life offers a strong point in favor of the “menage a trois” hypothesis: single domain NTTs are neither found in cyanobacteria nor in the vast majority of non-photosynthetic free-living eukaryotes. However, their transport activities are not unique. Besides NTTs, mitochondrial carrier family proteins are also known to transport nucleotides. Corresponding adenine nucleotide transporters have been identified in mitochondria, as well as in various other organelles [20,74,75]. Moreover, TAAC was identified as a thylakoid ATP/ADP carrier in *Arabidopsis thaliana* [76] and found to also transport phosphoadenosine 5′-phosphosulfate across the plastid envelope [77]; SLC35B1, a member of the solute carrier family was shown to represent the long-term sought ATP/ADP exchanger in the endoplasmic reticulum of eukaryotes [78]; and a new family of purine transporters that are not related to NTTs was discovered in microsporidia [79]. The host cell that gave rise to Archaeplastida, therefore, most likely already contained one or several metabolite transporters capable of adenine nucleotide translocation, and hence plastidic ATP/ADP exchangers could have also evolved via gene duplications of already existing eukaryotic transporters and functional divergence of the paralogues in a scenario without an additional gene donor. However, this did not happen; instead, of all transport proteins, NTTs somehow appeared so early in the process of plastid evolution that they are found in all photosynthetic eukaryotes. This seemingly gratuitous appearance of NTTs indicates that a non-cyanobacterial and non-eukaryotic gene donor who contained NTT genes might have been involved in the plastid establishment, without considering branching patterns of single gene phylogenetic trees.

The number and sequence of eukaryote-eukaryote symbioses that led to the complex plastids being found in the various groups of algae is currently debated [4,5,7]. Complex plastids are called secondary plastids if they evolved via endosymbiosis of an alga with primary plastids (a secondary endosymbiosis). In the case of complex plastids of red algal origin, the numbers of endosymbioses that are postulated range from just one secondary endosymbiosis, in one common ancestor of all groups of algae in which they are found (cryptophytes, haptophytes, stramenopiles, and alveolates) [80], to four secondary endosymbioses, assuming independent plastid gains in cryptophytes, haptophytes, stramenopiles, and alveolates [3]. Which scenario is most likely depends on the weighting of costs and probabilities that are associated with plastid gains versus plastid losses. With increasing sequence data available, and the discovery of organisms with “missing link” status, more balanced hypotheses, in which complex plastids are proposed to have been passed on in tertiary endosymbioses (endosymbioses in which the endosymbiont already contains a secondary plastid), have been proposed [7,8,9,81].

Similar to the telltale sign of a cryptic endosymbiosis during plastid establishment that the presence of NTT-type ATP/ADP exchangers in all photosynthetic eukaryotes offers, the presence of additional NTTs in diatoms also provides insights into the origin and spread of complex plastids of red algal origin. Diatoms are members of the stramenopiles, a group that contains photosynthetic as well as non-photosynthetic members. The photosynthetic stramenopiles form a monophyletic group, the Ochrophyta, which is a sister group to a non-photosyntetic monophyletic group of stramenopiles, the Oomycota (oomycetes) [82]. If the plastids of Ochrophyta would have been gained in a common ancestor of stramenopiles with cryptophytes, haptophytes or alveolates, the non-photosynthetic stramenopiles would have secondarily lost their plastids [82]. Due to the phylogenetic position of the oomycetes as a sister group of the Ochrophyta, this process must have had happened independently at least two times [82], which is at odds with the observation that loss of photosynthesis is not uncommon, while the loss of entire plastids is highly uncommon (reviewed by Oborník [7]). Furthermore, such secondary losses of plastids would likely leave traces in the genomes, in the form of genes that were acquired with the plastids, but that do not have functions in core photosynthesis. NTTs are examples of such genes/proteins. From this point of view, it is interesting to see that Major et al. [41] identified single domain NTTs that do not descend from common plant NTTs in a number of photosynthetic stramenopiles (not only diatoms), as well as in cryptophytes and haptophytes, but not in alveolates, and not in the non-photosynthetic groups of stramenopiles. This distribution of single domain NTT genes across the tree of life supports a common origin (direct via secondary or indirect via tertiary endosymbiosis) of stramenopile, haptophyte, and cryptophyte plastids, to the exclusion of alveolate plastids. Furthermore, the suspicious absence of single domain NTTs from non-photosynthetic stramenopiles would be more in line with a gain of plastids at the base of Ochrophyta, than with losses of plastids in the non-photosynthetic stramenopiles. Interestingly, oomycetes do possess NTT-related genes, in the form of fused NTT-HEAT-cNBD domains; these genes are, among eukaryotes, exclusively found in oomycets [41]. Major et al. [41] therefore suggested that the acquisition of these NTT fusion proteins was an early event in the evolutionary history of oomycetes. It might be speculated that this acquisition of NTT-domain containing proteins might not have occurred if the ancestor of oomycetes would have already possessed NTTs.

## 5. Biotechnological Applications of Diatom NTTs

**Figure 2:**
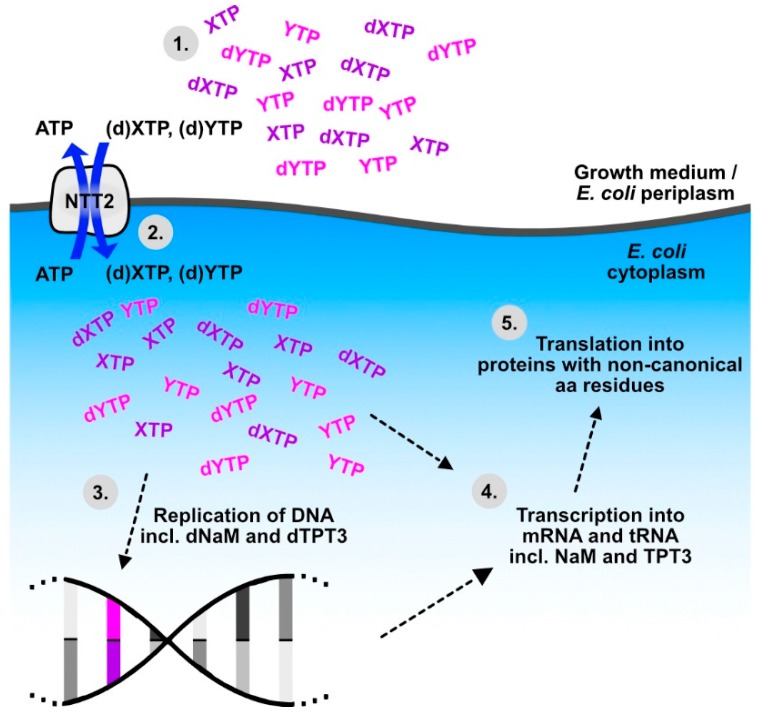
*Pt*NTT2 provides a crucial step to the workflow of expanding the genetic alphabet for the inclusion of non-canonical amino acids into proteins, as established by Zhang et al. [87]. (1) Artificial nucleotides (deoxy- and ribotriphosphates of NaM (dXTP, XTP) and TPT3 (dYTP, YTP)) are added to the surrounding medium. (2) The broad substrate specificity of *Pt*NTT2 allows for the uptake of (d)XTP and (d)YTP into *Escherichia coli*, in exchange for naturally occurring ATP. (3) DNA polymerase replicates templates that contain dNaM and dTPT3, leading to stable reproduction of the modified DNA (modified base pair in pink/purple). (4) Transcription of the modified DNA results in mRNA containing the NaM and TPT3. (5) Modified tRNAs, which fit codons with unnatural bases are transcribed and loaded with non-canonical amino acids, allowing the introduction of non-canonical residues into proteins.

For the experimental determination of the transport properties of NTT proteins, candidate transporters are overexpressed in *Escherichia coli*, and uptake or release of radiolabelled substrates can then be measured by incubating the NTT expressing *E. coli* cells with potential substrates [23,39,42,57,58,59,60,61,63]. In the course of these experiments, the *E. coli* cells take up the substrates of the tested NTTs, and thanks to the broad substrate spectrum of the *P. tricornutum* NTT2, this transport essay type of experiment has enabled a major breakthrough in synthetic biology: *Pt*NTT2 accepts not only the natural nucleotide substrates as tested in [39], but also synthetic nucleobases, which can form unnatural base pairs [83]. Such unnatural base pairs can be used to expand the genetic code to include codons for non-canonical amino acids, which can provide additional chemical functions to proteins (reviewed in [84,85]). *Pt*NTT2 allows the uptake of the artificial nucleosides dNaM and d5SICS in the form of triphosphates, which makes them accepted substrates for DNA polymerases and leads to their incorporation into DNA in vivo, when a template plasmid containing the unnatural base pair formed by d5SICS and dNaM has been added [83]. For the use of an unnatural base pair in a new codon, however, more prerequisites have to be met. For transcription into RNA, nucleosides are needed in the form of ribotriphosphates, a new tRNA gene with the complementary anticodon has to be introduced, and it needs to be loaded with the cognate amino acid [84]. After optimization of the *PtNTT2* gene sequence and expression system and a switch of the unnatural base pair to the better retained NaM-TPT3 [86], Zhang et al. [87] succeeded in integrating unnatural base pairs and non-canonical amino acids into the full replication-transcription-translation chain of biological information storage and retrieval (Figure 2). In their study, the unnatural base pair NaM-TPT3 was used in two different unnatural codons, for which tRNAs with cognate unnatural anticodons were introduced, which allowed the incorporation of natural or non-canonical amino acids into superfolder green fluorescent protein at the *E. coli* ribosome [87]. Such semi-synthetic expression systems have great potential in biotechnology and diatom NTTs continue to be part of such systems. Meanwhile, import efficiency is also considered as a factor in artificial nucleobase design [88].

In addition, also the NTT2 of *T. pseudonana* has a use in biotechnology, related to its capability to take up natural nucleotides (it has been tested for transport of d5SICS and dNaM triphosphates by Malyshev et al., but was found to not accept these substrates [83]). Pezo et al. [89] created *E. coli* mutants that are auxotrophic for deoxythymidine triphosphate (dTTP) and only survive due to the activity of *Tp*NTT2. Because the strains not only depend on the transgene (which is not found in wild type *E. coli*) but also on the supply of dTTP, this is a form of nutritional/trophic containment that can be used to prevent the spread of genetically modified strains. Furthermore, this also allowed optimization of the *Tp*NTT2 gene by directed evolution [89].

## 6. Conclusions and Outlook

A high diversity of NTT proteins can be found in diatoms. Some of these NTTs might have originated from intracellular bacteria independently from the gene acquisition that introduced the NTT-type ATP/ADP+Pi antiporter to the ancestor of photosynthetic eukaryotes. The various diatom NTTs show deviating transport properties, broader substrate spectra, and different intracellular locations, when compared to these typical plant NTTs. The diatom NTTs mediate net nucleotide provision to the plastid, which compensates for the lack of an oxidative pentose phosphate pathway and purine and pyrimidine biosynthesis pathways in diatom plastids.

Taken together, it is interesting to see that despite their unclear phylogenetic origins, NTTs facilitated at least three major breakthroughs, two in the evolution of life, and a third in synthetic biology: (i) the evolutionary establishment of primary plastids in all photosynthetic eukaryotes; (ii) the evolutionary establishment of complex plastids in diatoms; and (iii) the biotechnological development of semi-synthetic organisms, with expanded genetic coding capacity of their nucleic acids.

Transport proteins, including the not-yet-characterized diatom NTTs, play important roles in cellular physiology, as well as in the evolution of symbioses and organelles. The recent advances in organelle isolation [43], targeting prediction [46,55], electron microscopy [13], spectroscopy and biophysics [65], and genome editing techniques [90], which have become available for work with diatoms, open up opportunities to deepen our understanding of the intracellular exchange networks of energy, redox equivalents, and molecules such as nucleotides in these fascinating organisms.

## Figures and Tables

**Table 1:** Sequence properties, targeting predictions, and experimental localisations of diatom nucleotide transporters (NTTs).

Name	Protein ID ^1^	Targeting Prediction ^2^	ASAFAP Motif Identified by Ast et al. [39]	N-Terminal Elongation Identified by Major et al. [41]	Estimated Length ^3^	Estimated Size ^4^/kDa	Predicted Number of Trans-Membrane Helices ^5^	Experimental Localisations; Comments
*Pt*NTT1	49533	Plastid, high confidence	TEA-FAP (yes)	Yes	555	60.11	11	Targeted to the innermost plastid membrane via the stroma [39]; Ensembl identifier ^6^ Phatr3_J49533
*Pt*NTT2	45145	Not plastid, SignalP positive	ISA-TSS (no)	Yes	553	59.98	11	Plastid and nuclear envelope, ER cisternae, due to similarity with *Tp*NTT2, identical sub cellular localization was suggested [39]; Ensembl identifier ^6^ Phatr3_J11615
*Pt*NTT3	50189	Not plastid, SignalP negative	No signal peptide	Yes	666	73.05	11	Ensembl identifier ^6^ Phatr3_J50189; this model excludes the first nine residues
*Pt*NTT4	46794	Not plastid, SignalP negative	No signal peptide	Yes	591	66.42	8	Ensembl identifier ^6^ Phatr3_J46794
*Pt*NTT5	54110	Not plastid, SignalP negative	No signal peptide	Yes	547	58.32	7	Probably located in ER cisternae, C-terminus important for targeting [42]; Ensembl identifier ^6^ Phatr3_J54110
*Pt*NTT6	54907	Plastid, low confidence	VRA-LLP (yes)	Yes	557	61.47	7	Ensembl identifier ^6^ Phatr3_J54907; this model excludes the first 90 residues, and 35 residues of CDS in a postulated intron in the C-terminal region
*Tp*NTT1	26364	Plastid, high confidence	THG-FSP (yes)	Yes	525	57.44	11	Targeted to the innermost plastid membrane via the stroma [39], identified in plastid fractions via mass spectrometry [43]
*Tp*NTT2	24462	Not plastid, SignalP negative	SSA-EML (no)	Yes	632	69.53	10	Presequence not sufficient for plastid targeting, full-length fusion protein might be located in plastid membrane [39]
*Tp*NTT3	270249	Not plastid, SignalP positive	TEA-ALP (no)	Yes	692	75.17	10	Identified in plastid fractions via mass spectrometry [43]
*Tp*NTT4	270251	Not plastid, SignalP negative	No signal peptide	Yes	678	75.22	9	
*Tp*NTT5	9770	Not plastid, SignalP negative	No signal peptide	Yes	838	91.81	8	
*Tp*NTT6	24709	Not plastid, SignalP positive	SLA-HQH (no)	Yes	548	59.21	8	
*Tp*NTT7	270255	Not plastid, SignalP negative	No signal peptide	Yes	544	58.60	8	
*Tp*NTT8	270253	Not plastid, SignalP negative	No SP	Yes	589	63.59	10	

^1^ Protein IDs refer to the United States Department of Energy Joint Genome Institute (JGI; https://jgi.doe.gov/) genome portal [44] and sequencing projects for *P. tricornutum* [36] and *T. pseudonana* [34], as annotated in [39]. ^2^ SignalP v. 3.0 [45] in conjunction with ASAFind [46] was used to predict plastid targeting signals. Furthermore, all sequences were analysed with TargetP-2.0 [47]; here, the results of SignalP were confirmed and no mitochondrial transit peptides were predicted in any of the NTTs. ^3^ SignalP predicted signal peptides were removed; in case of ASAFind positive sequences, further 20 residues for estimated transit peptide domains were removed. ^4^ Molecular weights of the estimated mature proteins were calculated with the Expasy Compute pI/Mw tool (https://web.expasy.org/compute_pi/) [48]. ^5^ Transmembrane helices of the estimated mature proteins were predicted with TMHMM Server v. 2.0 (http://www.cbs.dtu.dk/services/TMHMM/) [49,50]. ^6^ Ensembl identifiers refer to the Phatr3 genome annotation of *P. tricornutum*, accessible via Ensembl Protists (https://protists.ensembl.org/Phaeodactylum_tricornutum/Info/Index).

**Table 2:** NTTs with unusual activities compared to ATP/ADP + Pi antiporters (like the plant NTTs shown in Figure 1a).

Group of Organisms	Species/Strain	Name	Activity	Role	References
Diatoms	*Thalassiosira pseudonana*	NTT1	Symport of ADP or ATP with protons	Net import of full set of nucleotides into the plastid	[39]
		NTT2	Exchange of (d)NTP against ATP		
	*Phaeodactylum tricornutum*	NTT1	Symport of ADP or ATP with protons		
		NTT2	Exchange of (d)NTP against ATP		
		NTT5	Exchange of GTP, GDP, dATP or dGTP against ATP or ADP	Provision of GTP, GDP, dA/GTP and chemical energy to the ER lumen	[42]
*Chlamydiae*	*Chlamydia trachomatis* ^1^	Npt2Ct	Symport of ATP, CTP, UTP and GTP with protons	Net import of full set of ribo-nucleotides into the symbiont	[57]
	*Protochlamydia amoebophila* UWE25 ^2^	NTT2	Antiport of UTP, ATP, GTP and CTP	Net import of ribo-nucleotides and NAD^+^ into the symbiont	[58]
		NTT3	Symport of UTP with protons		
		NTT4	Exchange of NAD^+^ with ADP		
		NTT5	Symport of GTP or ATP with protons		
Microsporidia	*Trachipleistophora hominis*	NTT1, NTT2, NTT3	Antiport of ATP/GTP against ADP/GDP	Energy parasitism, transport of NAD^+^ and NADH	[59,60]
		NTT4	Symport of GTP or ATP with protons	Net import of purine nucleotides	

^1^*Chlamydia trachomatis* additionally has an ATP/ADP + Pi antiporter (Npt1Ct) [57]. ^2^ *Protochlamydia amoebophila* strain UWE25 additionally has an ATP/ADP + Pi antiporter (NTT1) [58,61].
